# Integrated chronic care models for people with comorbid of HIV and non-communicable diseases in Sub-Saharan Africa: A scoping review

**DOI:** 10.1371/journal.pone.0299904

**Published:** 2024-03-15

**Authors:** Rumbidzai Chireshe, Tawanda Manyangadze, Keshena Naidoo

**Affiliations:** 1 Discipline of Public Health Medicine, College of Health Sciences, University of KwaZulu-Natal, Howard Campus, Durban, South Africa; 2 Department of Geosciences; School of Geosciences, Disasters, and Development, Faculty of Sciences and Engineering, Bindura University of Science Education, Bindura, Zimbabwe; 3 Department of Family Medicine, School of Nursing and Public Health, University of KwaZulu Natal, Howard Campus, Durban, South Africa; University of Ghana College of Humanities, GHANA

## Abstract

**Background:**

Integrated health care is an approach characterized by a high degree of collaboration and communication among health professionals. Integration of HIV/NCD is recommended to enhance the quality of healthcare services being provided. Duplication of limited resources is minimized, and a holistic care approach is promoted by shifting from acute and reactive care to care that embraces patient-centredness that includes promotive health and disease surveillance. The high burden of HIV disease in sub-Saharan Africa (SSA) combined with the increasing prevalence of chronic non-communicable diseases (NCDs) necessitates a review of how health systems has been doing to deliver quality integrated care for people living with HIV (PLWH) and comorbid chronic NCDs.

**Methods:**

A scoping review was conducted to identify and describe all publications on integrated chronic care management models at the primary care level in the SSA context, particularly those that addressed the care of PLHIV with co-morbid chronic NCDs. The inclusion and exclusion criteria were applied, and duplicates were removed.

**Results:**

A total of twenty-one articles were included in the final review. Integrated healthcare systems were reported in only eight SSA countries–(South Africa, Uganda, Kenya, the United Republic of Tanzania, Zambia, Malawi, Zimbabwe and Swaziland). Integrated care systems adopted one of three health models. These included added-on NCD services to previously dedicated HIV care facilities, expansion of primary care facilities to include HIV care and establishment of integrated care services. Short-term benefits included staff capacitation, improved retention of patients and improved screening and detection of NCDs. However, the expansion of existing services resulted in an increased workload with no additional staff. A significant positive change noted by communities was that there was less or no stigmatisation of people living with HIV when attending dedicated HIV clinics.

**Conclusion:**

Evidence of integrated healthcare services for PLWH and co-morbid of NCDs in SSA is scanty. Data on some short-term benefits of integrated care was available, but evidence was absent on the long-term outcomes. Randomized clinical trials with clearly defined comparator groups and standardized measures of HIV and NCD outcomes are needed to demonstrate non-inferiority of integrated against non-integrated care.

## Background

The increasing burden of comorbid HIV infection and chronic non-communicable diseases (NCDs) such as hypertension and diabetes in Sub-Saharan Africa (SSA) necessitates a review of how healthcare services can best provide integrated care for people with chronic multi-morbidities [1]. The successful roll-out of antiretroviral treatment has transformed HIV from a terminal illness into a chronic disease [2]. People living with HIV (PLHIV) are now living longer with an increased risk of developing other chronic non-communicable diseases (NCDs) [3–7]. The increasing prevalence of people with both HIV and chronic NCD co-morbidity presents a challenge to primary healthcare systems in SSA that mostly focus on single disease management and acute episodic care [8, 9]. It is unclear how health systems in the SSA region can best provide integrated and quality health care to patients with multiple chronic health conditions.

Integrated health care is an approach characterized by a high degree of collaboration and communication among health professionals [10]. Multi-morbidity is the presence of two or more chronic health conditions in the same individual, and it presents complexity in the medical management of such patients [11]. Most of the current health systems and standard treatment guidelines in SSA focus on single disease management and do not facilitate the coordination of care for patients with multi-morbidities [12]. This can result in polypharmacy, duplication of care, and increased healthcare expenditure [8]. Integrated care management, as advocated by the World Health Organisation, aims to provide comprehensive care that will increase healthcare utilization as well as promote self-management among patients [12]. There has been progress in several African countries towards HIV and NCD service integration. However, further evaluation of the integrated HIV and NCD programmes is needed to provide insight into the associated benefits and risks, and to guide future health system development [13].

The goal of integration of care is to enhance the quality of care and quality of life, patient satisfaction, and system efficiency for people by cutting across multiple services, providers, and settings [14]. There are several resource gaps in resource-limited settings such as SSA, evidence on integrated care having a potential to be cost-effective and improve delivery of services to patients remains scanty. Evidence on health models for integrated care emanates mostly from high-income countries (HIC).

This scoping review aimed to describe the extent to which the integrated chronic care delivery has been adopted in SSA and which models of integrated care delivery are most and least prevalent in this region, in particular those that address the care of PLHIV with co-morbid chronic NCDs [12]. Therefore, this study identified the different health models that have been described in published literature prevalent in SSA for integrated chronic care and explores barriers and facilitators to integrated care systems in the sub-Saharan African region. This review also highlights gaps in evidence on integrated care models in SSA.

## Methods

### Study design

A scoping review was conducted to identify, map, and describe the evidence on integrated care models for people with comorbid HIV and non-communicable diseases in Sub-Saharan Africa. Arksey and O’Malley’s methodological framework was used to guide this process [15]. The framework involves (i) identifying the research question, (ii) identifying relevant studies, (iii) study selection, (iv) charting the data, and (v) collating, summarising, and reporting results.

#### Identifying the research question

The major research question was “’How are primary health care systems in SSA delivering integrated care to people living with HIV and chronic NCD co-morbidities?”

Research sub-questions are as follows:

i. How are primary health care systems in SSA adopting to the integrated health care delivery for people living with HIV and chronic NCD co-morbidities?
ii. How widespread is the delivery of integrated health care for PLHV and NCD co-morbidities in SSA?
iii. What is the reported impact of the integrated care models implemented on patient and public health outcomes in SSA?

#### Identifying relevant articles

The search strategy was developed as a broad framing of the population (people with HIV and chronic NCD co-morbidity), concept (integrated chronic care), and context (health care settings in SSA).

To identify relevant studies, the authors performed a keyword search in the following electronic databases: PubMed; Cochrane Library, SCOPUS, Google scholar; EBSCOhost platform (Academic search complete, Health Source: Nursing/Academic Edition, CINAHL, PsycINFO and MEDLINE), web of Science from January 2010 (to make sure that the studies used gives us meaningful up-to-date data) to January 2022. Keywords such as “patients”, “people”, “HIV”, “non-communicable disease”, “comorbidity”, “multi-morbidity”, “multimorbidity”, “integrated care”, “models”, “health outcomes”, “SSA” were combined to form a search strategy. Use of symbols and connectors plus Boolean terms (AND/OR) and medical subject headings such as non-communicable diseases, integrated care and HIV were used to refine the search strategy (Supplementary file 1). The study design limitation was removed during the search. An experienced librarian was involved during the development of the search strategy, searching for the articles, and de-duplication of the search results. The World Health Organization website as well as the references of the included articles were also searched for relevant models, the focus was directed on the integration of chronic disease care/management into health systems. 3.

### Study selection

**Fig 1:**
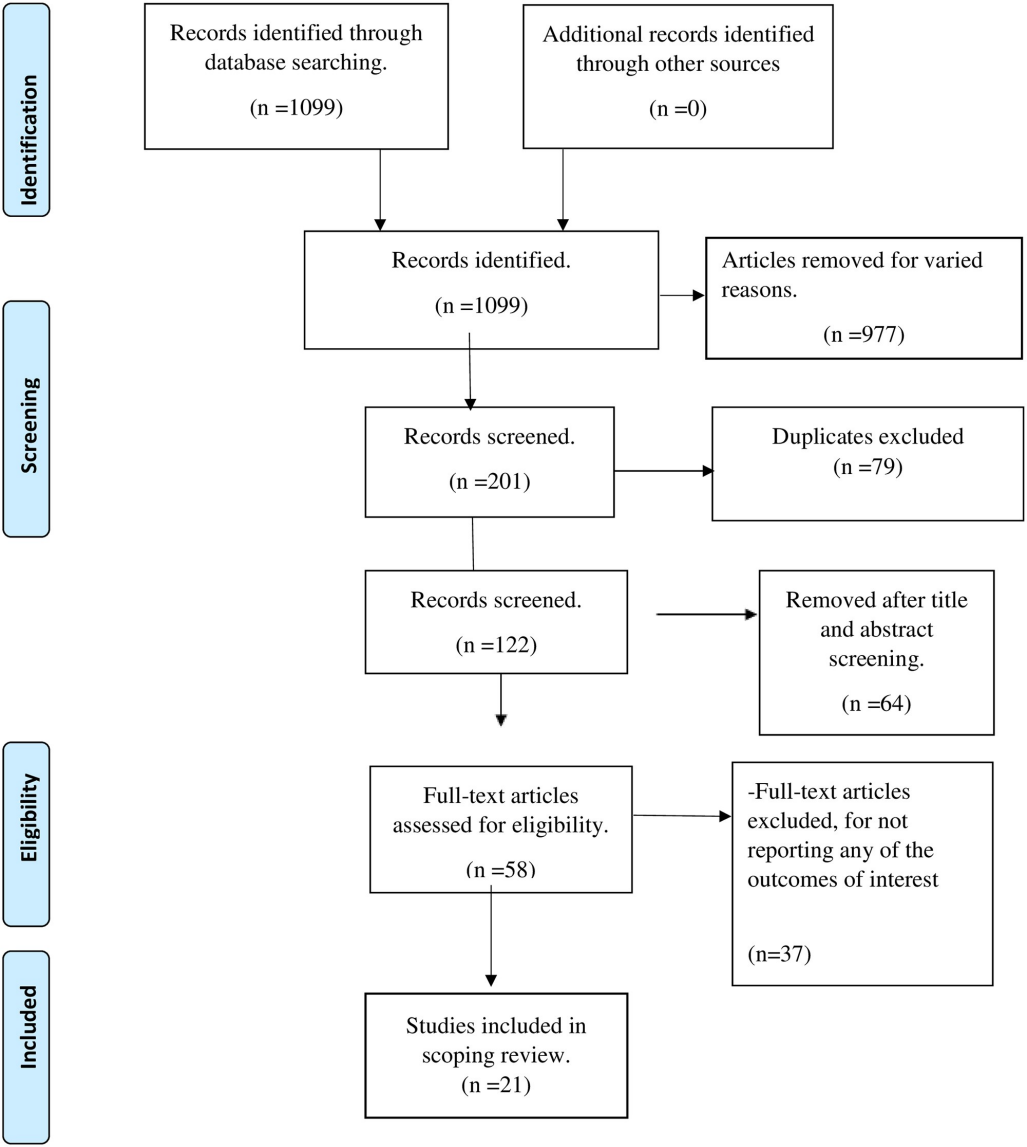
PRISMA flowchart.

After the title screening, the included studies were exported to the EndNote Library and duplicates removed. Two reviewers independently conducted the titles, abstract, and full-text screening using the study’s eligibility criteria as a guide. Discrepancies that arose at the abstract screening stage were resolved by discussion; and at the full-text screening stage, a third reviewer was engaged to address all discrepancies. The University of KwaZulu-Natal Library services was used to retrieve full-text articles not freely available online. The PRISMA flow chart (Fig 1) was used to report the screening results. The eligibility and inclusion criteria are as explained below:

### Inclusion criteria

We included original research articles reporting on the integration of healthcare for patients with HIV and chronic NCDs in SSA countries, articles utilising any study design addressing the research question, all articles in English or (English translation) and published from January 2010 till January 2022 were included.

## Exclusion criteria

Articles that were not original and non-English were excluded, and articles that were not relevant to the main subject were also excluded from the study, this included articles that did not have full texts.

### Charting the data

Data from the studies that were used in this article was extracted using an extraction form, which included the following data: author and year, study population, study design, study setting, relevant findings, significant findings, and conclusion.

### Collating, summarising, and reporting the results

The data extracted from the included studies were analysed for emerging themes and were critically examined about the main review question. The data was collated and analysed using a thematic content analysis approach. NVIVO software version 10 was used to code the data from the included studies [16]. The process below was followed:

- Coding data from the included articles
- Categorizing the codes into major themes
- Displaying the data
- Identifying key patterns in the data and identifying sub-themes.
- Summarising

## Results

### Study selection

The initial search yielded 1099 publications, of which only 201 publications were eligible for the study, 122 remained after 79 duplicates were removed (see supplementary file 1). After title and abstract screening, 58 studies underwent full-text screening. Twenty-three of these studies were excluded as they did not report on evidence of integrated chronic care management models at the primary care level in SSA, particularly people living with HIV and NCD co-morbid, eleven were excluded because of different reasons that included the type of publication of the studies and two were not from SSA. Hence twenty-one studies were included in this review, as outlined in the PRISMA flow diagram (Fig 1).

### Characteristics of the included studies

The included articles were conducted in SSA and published between 2010 to 2022.

Twenty-one articles were identified: seven in South Africa (33%); five in Uganda (24%), three in Kenya (14%), two in United Republic of Tanzania (9%), and one in Zambia, Malawi; Zimbabwe; and Swaziland (5% each).

The scope of integrated services varied widely between programs, with some only integrating screening services while others provided treatment for multiple NCDs and HIV. Most of these programs nearly achieved the scope of services provided under chronic care management (CCM).

**Fig 2:**
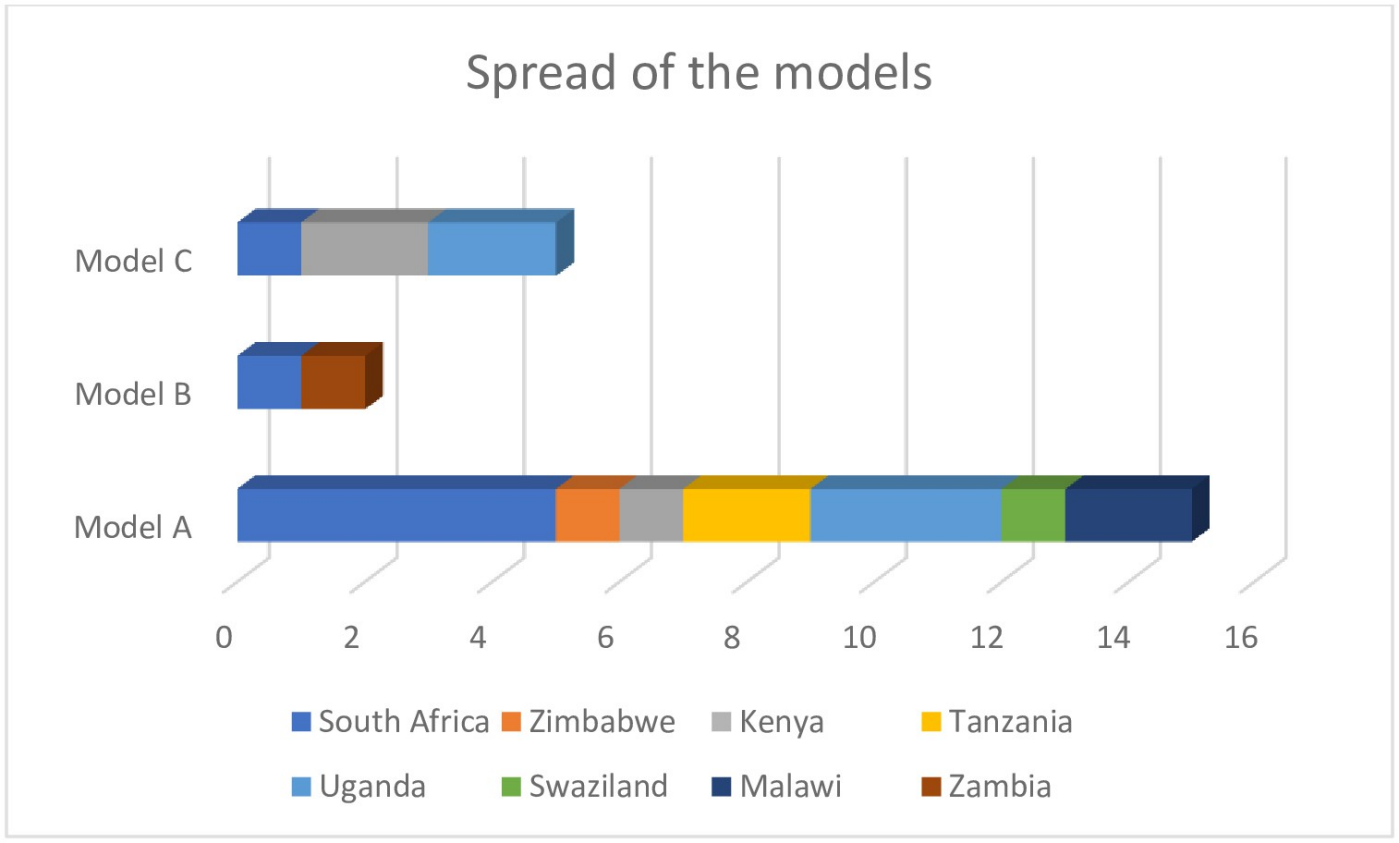
Spread of models *(own work)*. Note: Graph produced from raw data. Own work.

There were seven (7) cross sectional and longitudinal mixed methods studies from South Africa, Nigeria, Zambia, Uganda and Malawi, three (3) cross-sectional descriptive studies from Kenya, Tanzania and Uganda. Three (3) retrospective studies from South Africa, Uganda and Malawi, two (2) qualitative studies from South Africa and Kenya. One (1) prospective cohort study from Tanzania and Uganda, one (1) programmatic description study from Zimbabwe, one (1) interrupted time series study from South Africa, one (1) quasi-experimental study from South Africa, one (1) community health campaign study from Uganda and one (1) population-based study from Kenya found in this paper (Fig 2).

The study identified these models in 8 different countries in SSA, with South Africa having the highest number of studies included in the study five studies that adopted model A, one adopted model B and one model C. Zimbabwe had one study that adopted model A, whereas Kenya had 3 studies in this study, one that adopted model A and two that adopted model C. Tanzania and Uganda had one study that included both countries in its study setting, Tanzania had 2 studies selected for this study both adopted model A. Uganda had a total of five studies, three adopted model A and two adopted model C. Swaziland and Zambia each had one study which adopted model A, Zambia also had one study that adopted model B (Fig 2).

### Adoption of the integrated health care delivery for people living with HIV and chronic NCD co-morbidities in SSA health systems

There were several barriers and facilitators to -adoption of NCD/HIV integration in SSA health systems as highlighted in the studies. The barriers include a lack of functional equipment e.g. BP machines, inadequate supply of pre-packed medicines and medicines in general, and additional workload to providers for healthcare services [19]. Ameh et al., 2020 cited staff shortage and work overload as a barrier to the integration of HIV/NCDs [18], other barriers included system barriers, lack of economic resources, and other resources. Continuity of care, flexibility of care and destigmatization were some of the facilitators to integration. In some studies, staff training and the provision of prepacked medicines were also examples of facilitators of NCD/HIV integration.

### Impact of integrated care models for HIV and NCDs on patient health outcomes

There were multiple positive outcomes described in integrated care models (Tables 1–3). Some studies reported improved blood pressure and glycaemic control after just six months of implementation [35]. Retention in care was also linked to high levels of viral suppression in PLWH. A study carried out in Uganda, the population-based risk for cardiovascular disease in areas that implemented integrated chronic care services was significantly lower than in areas that did not [27]. Several studies reported increased client satisfaction as integrated services helped destigmatize HIV and reduced the number of clinic visits [17, 20]. Community screening identified new patients and facilitated the linkage of care [20].

**Table 1:** Summary of studies on model A: NCD services integrated into centres originally providing HIV care.

Country, program	Authors & year	Site of care	Services offered	Study Design	Study population	Number of sites	Reported impact or outcomes
Kenya, AMPATH [17]	Osetinsky et al. 2019	Primary care clinics originally providing HIV care treatment	Hypertension, and HIV screening and treatment	Cross-sectional study	Patients with HIV and/or NCDs	-	Increased Hypertension controls. Increased retention in care and there was co-location of services.
South Africa, ICDM [18]	Ameh et al. 2020	Primary clinics originally providing HIV care treatment	Hypertension and HIV screening and treatment	Longitudinal mixed method study	Patients with HIV and/or NCDs	-	suboptimal quality of care in five of the eight priority dimensions of care used as leverage for the NCD program. Increased waiting time, reduction in stigma due to non-segregation, staff shortage, work overload
South Africa, ICDM [19]	Ameh et al. 2017	Primary clinics originally providing HIV care treatment	NCDs and HIV screening and treatment	Longitudinal mixed method study	PLWHIV and NCDs	7 PHC facilities	Reduced facility-related stigma. Malfunctioning blood pressure machine, irregular pre-packing of drugs, long waiting times.
Tanzania and Uganda, MOCCA [20]	Birungi et al. 2021	Primary health care facilities providing HIV care treatment	Hypertension, diabetes, and HIV screening and treatment	Prospective cohort study	Patients with hypertension, diabetes, and HIV	10 PHC facilities	High levels of retention in care were reported, 2640 patients were screened of whom 2273 (86%) were enrolled into integrated care (832 with HIV infection, 313 with diabetes, 546 with hypertension, and 582 with multiple conditions).
Zimbabwe, Nurse-led DM, and HPT model [21]	Frieden et al. 2020	Primary health care facilities and hospitals providing HIV care treatment	Diabetes, hypertension, and HIV screening and treatment	programmatic description study	Patients with diabetes, hypertension, and HIV	7 PHCs and 1 hospital	Sufficient knowledge and skills acquired by nurses. 3094 patients were screened and registered to the program, 188 with diabetes only, 2473 with hypertension only, and 433 with both diabetes and hypertension. There was high retention in care although there was pressure on services because of the provision of free medication
South Africa, Integration Clubs [22]	Gausi et al. 2021	Primary health care facilities providing HIV care treatment	NCDs and HIV screening and treatment	Retrospective cohort study	Patients with NCDs and HIV	2 PHC clinics	247 HIV-infected patients enrolled, 221 (89.5%) had hypertension, 4 (1.6%) had diabetes mellitus and 22 (8.9%) had both diseases. High retention in care, increased efficiency in terms of optimizing utilization of resources, adherence was maintained
South Africa, Shippee’s Cumulative Complexity Model (CCM) [23]	Matima et al. 2018	Community health centre providing services to both HIV and diabetic patients	Diabetes and HIV screening and treatment	Exploratory qualitative study	Patients with Diabetes and HIV	1 Community health care centre	Self-care-related workloads were largely around nutritional requirements, pill burden, and stigma, power mismatch between patients and healthcare workers
Uganda, HIV/ HPT care cascade model [24]	Muddu et al. 2019	Primary health care facilities providing HIV care treatment	HIV and Hypertension screening and treatment	Retrospective cohort study	Patients with hypertension and PLWHIV	3 high volume HIV clinics	High retention in care, of 1649 enrolled patients, 98.5% were initiated on HIV treatment, 456 (27.7%) participants were screened for HTN, of whom 46.9% were diagnosed, 88.1% were initiated on treatment,
Malawi, a collaboration between Centres for Disease Control and Prevention (CDC) partnered with Malawi’s Ministry of Health (MOH) and the Lighthouse Trust [25]	Patel et al. 2018	Primary health care facilities providing HIV care treatment	HIV and Hypertension screening and treatment	Cross sectional mixed method study	Patients with hypertension and PLWHIV	2 high volume HIV clinics	Improved monitoring and evaluation, positive outcomes for HIV/HPT patients, and development of a standard protocol for treatment
Swaziland, HIV/ CVDRF (Cardiovascular disease rheumatoid Factor)model [26]	Rabkin et al. 2018	Primary health care facility providing HIV care services	HIV and cardiovascular risk factor screening	Cross sectional mixed method study	PLWHIV	1 HIV clinic	High prevalence was reported, Screening was feasible, exposed system barriers that can impede services, there was work overload on health care workers
Uganda, NCD/HIV integration model [27]	Sando et al. 2020	Primary health care facilities providing HIV care services	NCDs and HIV Screening and treatment	Cross-sectional descriptive study	Patients with HIV and NCDs	-	A decline in CVD risk leads to a decline in overall cost
Tanzania, Models of Chronic Care in Africa (MOCCA) [28]	Shayo et al. 2021	Primary health care facilities providing HIV care services	NCDs and HIV screening and treatment	Cross-sectional descriptive study	Patients with HIV and NCDs	5 health facilities	Positive attitude towards the model, High acceptability, high satisfaction, multi-condition education, patient time and cost saving, and early detection of diseases
Malawi, Integrated Chronic Care Clinic (IC3) [29]	Wroe et al. 2020	Primary health care facilities providing health services and HIV care services	NCDs and HIV screening and treatment	Retrospective cohort study	Patients with HIV and NCDs	14 PHC clinics	Significant improvements in NCDs, facilitated rapid decentralization and access to NCD services in Malawi, high retention in care
South Africa, ICDM [30]	Ameh et al. 2017	Primary clinics originally providing HIV care treatment	NCDs and HIV screening and treatment	Interrupted time series study	PLWHIV and NCDs	435 pilot facilities and 433 PHC facilities for comparison	The pilot facilities had a 6% greater likelihood of controlling patients’ CD4 counts than the comparison facilities (coefficient = 0.057; 95% confidence interval: 0.056 to 0.058; P, 0.001). The model had a small effect in controlling patients’ CD4 counts and BP

Model A integrated clinics reported improved different health outcomes such as linkage, retention, and adherence but there was no findings/evidence on clinical benefits in other studies Ameh et al 2017.

**Table 2:** Summary of studies on Model B: HIV care integrated into primary health care already offering NCD services.

Country, program	Authors & Year	Site of care	Services offered	Study design	Study population	Number of sites	Key process indicators or outcomes
South Africa, HIV/NCD integration model [31]	Rawat et al. 2018	Primary healthcare facilities providing services for NCDs	NCDs and HIV screening and treatment	Quasiexperimental design	Patients with NCDs	131 PHC clinics	Increase in people accessing ART in PHCs from 1614 to 57, 958, however, this compromised patients on hypertension care
Zambia, Centre for Infectious Disease Research in Zambia (CIDRZ) [32]	Topp et all. 2013	Primary healthcare clinics provide general outpatient department services	HIV, and NCD services offered during routine primary healthcare visits	Cross sectional mixed methods study	All patients	12 PHC clinics	Successful integration of HIV testing, care, and treatment into outpatient services in Lusaka Zambia Integration increased efficiency of clinic space, resources, and staff time increased HIV case finding, and decreased HIV-associated stigma also found

Model B clinics had the advantage of destigmatizing HIV. PLWH was seen as any other patient with chronic disease. However, the patient burden was reported to increase as new patients identified with HIV infection were added to the existing client numbers. There was no mobilization of additional resources to accommodate the increased workload for facility staff, which potentially compromised the quality of care for patients.

**Table 3:** Summary of studies on Model C: Simultaneous introduction of integrated HIV and NCD services.

Country, program	Authors	Site of care	Services offered	Study design	Stud population	Number of sites	Key process indicators or outcomes
Uganda, Kakyerere community health campaign [33]	Chamie et al. 2012	Sites temporarily providing different health services including HIV care screening	HIV, TB, malaria, diabetes, and hypertension screening but no treatment	Community health campaign	Everyone from the community	3 sites	The high outcome in testing, a high burden of undiagnosed HIV, hypertension, and diabetes reported and there was a provision of free medication
South Africa, mobile testing unit [34]	Govindasamy et al. 2013	A mobile unit providing screening	HIV, TB, diabetes, and hypertension screening	Cross sectional mixed methods study	Everyone from the community	1 mobile unit	Above half linked to care, the yield of new diagnoses was HIV (5.5%), TB suspects (10.1%), diabetes (0.8%), and hypertension (58.1%). care was run during the day, so it was missed by a lot of the working class.
Uganda, SEARCH [35]	Kwarasiima et al. 2019	Sites temporarily providing different health services including HIV care screening	HIV and Hypertension screening together with other conditions	Longitudinal mixed methods study	Everyone from the communities	10 communities	Linkage to care within one-year, improved hypertension controls and there was the prevention of redundant clinic visits
Kenya, Medication Adherence Clubs [36]	Venables et al. 2016	Primary health care facility providing health services and HIV care services	HIV, and NCD services offered during routine primary healthcare visits	Qualitative study	Patients with HIV and NCDs	1 PHC clinic	High acceptability only for members of the club, reduced clinic visits, health education, and de-stigmatization of HIV-positive patients
Kenya, Hybrid HIV/NCD Integration model [37]	Kasaie et al. 2020	Primary health care facilities providing health services and HIV care services	NCDs and HIV screening and treatment	Population-based study	Patients with HIV and NCDs	3 health regions	Integrated HIV/NCD intervention will reduce costs on patients and health systems in the long run

The main advantage of this model was the convenience to patients. Patients identified through screening required fewer clinic visits to access care for their chronic diseases, thereby reducing health expenditure.

Organizational and managerial advantages were also identified, including more efficient use of staff time and clinic space, improved teamwork and accountability, and more equitable delivery of care to HIV and non-HIV patients [32]. However, integration did not solve ongoing human resource shortages or inadequate infrastructure, which limited the efficacy of the model and were perceived to undermine service delivery [32].

Medication adherence clubs that were established in Kibera, Kenya saved time, prevented unnecessary queues in the clinic, and provided people with health education and group support whilst they collected their medication. Some patients and healthcare workers felt that medical adherence clubs reduced stigma for HIV-positive patients by treating HIV as any other chronic condition [36].

Integration clubs in Cape Town, South Africa reported enhanced adherence to chronic treatment. This was accompanied by improved HIV viral suppression among patients. [22]. Screening of patients, resulted in high retention in care, increased efficiency in terms of optimizing utilization of resources, and adherence was maintained. This also helped with improved blood pressure and glycaemic control [22]. In Lusaka, Zambia the implementation of an integrated chronic care model substantially changed the organization of service delivery across a range of clinic systems [32].

## Linkage of care

Thirty-nine percent of newly HIV-diagnosed patients were linked to care in Uganda. Sixty-five percent of hypertensive patients and 23% of diabetic patients were diagnosed, and 43% and 61% of these patients were linked to care, respectively [33].

## Discussion

This scoping review aimed to describe the extent to which the integrated chronic care delivery has been adopted in SSA and which models of integrated care delivery are most and least prevalent in this region, particularly those that address the care of PLHIV with co-morbid chronic NCDs. The review identified limited evidence on integrated chronic care health models incorporating care for PLWH and chronic NCD co-morbidities in SSA, with only eight SSA countries represented in this review. Three models of NCD and HIV healthcare integration in the SSA region were described. Most studies (n = 14 or 67%) reported on dedicated HIV services that incorporated screening and/or treatment for chronic NCDs. Fewer studies (n = 2 or 9%) reported HIV services being introduced into existing PHC services, and only five studies (24%) described models providing both HIV and chronic NCD services from inception.

There were multiple positive effects reported regarding integrated chronic care services. De-stigmatization of HIV was one of the most reported positive outcomes that came from restructuring existing primary health facilities to include HIV services [36]. By offering screening services for both HIV and chronic NCDs in the same facility there was a reported increase in case finding and possible early diagnosis of chronic conditions. Early detection and treatment of chronic diseases are associated with reduced morbidity and mortality from cardiovascular events [27]. However, there was no data from the included studies on the long-term effects of integrated care on morbidity and mortality. Facilities that practised integrated care delivered comprehensive care to patients thereby reducing the need for multiple clinic visits for individual health conditions. [35]. Patient care was also enhanced through more frequent assessments of weight and blood pressure as well as improved attention to adherence to therapy. A study carried out in Cape Town noted increased utilization of health services, improved adherence to chronic medication and better overall control of chronic illnesses [22].

The staff at facilities that adopted integrated chronic care systems benefited from additional training and mentoring [21]. However, there was no increase in the number of clinical staff despite an increased workload. An initial increase in patient numbers was reported in the period following the provision of integrated services due to the convergence of clients from two or more pre-existing departments. However, the number of patient visits will likely decrease in the future as multiple health concerns will be addressed at one visit compared to several disease-specific clinic visits [21].

By expanding on established policies and protocols developed for dedicated ART programmes health policymakers can improve the current primary care services available to patients with multi-morbidities [38]. Pharmacological management of patients with chronic multi-morbidities is problematic in many SSA countries where supply chains are unreliable, and patients must pay for certain medications [21]. For sustainable and effective delivery of integrated chronic care services it is integral to find solutions to prevent drug supply interruptions [21]. Many primary care facilities, especially in rural locations have limited access to laboratory and x-ray services. As a result, comprehensive care for patients is compromised as patients will need to be referred to other centres for specific services [32, 33].

The models presented are most often based upon project-based initiatives which are donor-dependent and thus should be considered by program planners in this light [21]. As LMIC address the growing NCD epidemic among PLWHIV, program planners and policymakers might need to look at the HIV response to inform NCD response. There is a need for a change in the mindset of health professionals and health policymakers to a more person-centred approach. This should ideally start with health professions training and the primary healthcare services adopting an integrated approach to caring for people with chronic co-morbidities.

Our review has several limitations. First, this scoping review used a simple search strategy, and a highly sensitive search strategy may have identified more articles addressing the research question. Furthermore, the review did not compare clinical outcomes for HIV and NCD in patients receiving integrated versus those not receiving integrated models of care, there is a need for randomised controlled trials that compare clinical outcomes. This scoping review was also limited to articles published in English language or translated to English, which might have introduced selection bias and limited retrieval of relevant studies published in other languages.

While evidence for integration of NCD/HIV programs is scarce, in that lies the potential to address the chronic care needs of those suffering from NCDs. Through the use of HIV program approaches, such as defaulter tracing; use of community engagement; adapted tools and systems, including longitudinal medical records to ensure continuity and coordination, optimized supply chain management; and referrals and linkages, sustainable solutions for reducing NCD-related morbidity may be discovered [32].

## Conclusion

Although there is scanty evidence from the SSA region on successful integration of primary healthcare services for people with HIV and comorbid chronic NCDs. One important factor for health service delivery is the ability to use the current HIV infrastructure to treat multimorbid patients for NCDs without compromising the quality of care. This is especially true in high-HIV-burden settings where NCD comorbidity is rising, and the epidemiological transition is occurring quickly. Integrated chronic care management models were identified in only eight SSA countries. The approach to providing integrated care to people with HIV and comorbid NCDs in SSA varied greatly with no coherent model addressing comprehensive care involving screening, treatment, and secondary prevention.

There was evidence of improved linkage to care, adherence to therapy and patient satisfaction with integrated chronic care, however, there is limited data on long-term benefits. Current evidence on integrated chronic care management systems in SSA supports widespread adoption of integrated chronic care services at the primary care level. However, there is no standardised model for providing integrated care.

## Recommendation

Appropriate allocation of resources should be considered by ministries of health to support the delivery of quality integrated care for PLWH and co-morbid chronic NCDs. Reassignment of staff and resources from standalone service departments to primary care facilities providing integrated care will alleviate some of the pressures of increased patient numbers. In addition, external funders such as non-governmental organisations involved with antiretroviral therapy (ART) services can assist with resources and clinical expertise. The standard of care provided to HIV patients at NGO-run facilities are reportedly higher due to additional resources and accountability to funders. It is thus of value to explore private-public partnerships in strengthening existing infrastructure.

To prove that integrated care is not inferior to non-integrated treatment, randomized clinical trials with well-defined comparator groups and standardized measurements of HIV and NCD outcomes are required.

## Limitations

This scoping review used a simple search strategy, and a highly sensitive search strategy may have identified more articles addressing the research question. Furthermore, this scoping review was limited to articles published in English language or translated to English, which might have introduced selection bias and limited retrieval of relevant studies published in other languages.

## Supporting information

S1 File Database searches. (DOCX)

S1 Checklist PRISMA 2020 checklist. (PDF)

## Abbreviations

AMPATH: Academic Model Providing Access to Healthcare
CVD: Cardiovascular disease
DALY: Disability-adjusted life year
DM: Diabetes mellitus
HIV: Human Immuno-deficiency virus
HTC: HIV testing and counselling
HTN: Hypertension
IC3: Integrated chronic care clinic
LMICs: Low-and Middle-Income countries
MESH: Medical Subject Headings
MMAT: Mixed Method Appraisal Tool
MSF: Médecins Sans Frontières
NCDs: Noncommunicable diseases
PCC: Population Content Context
PHC: Primary healthcare
PIK: Partners in health
PLWHIV: People living with HIV
PRISMA-P: Preferred Reporting Items for Systematic Reviews and Meta-Analysis Protocols
SSA: sub-Saharan Africa
UKZN: University of KwaZulu-Natal
UN: United Nations
WHO: World Health Organization

## References

[1] Peer N, de Villiers A, Jonathan D, Kalombo C, Kengne A-P. Care and management of a double burden of chronic diseases: experiences of patients and perceptions of their healthcare providers. PloS one. 2020;15(7):e0235710. doi: 10.1371/journal.pone.0235710 32673339 PMC7365408

[2] Taylor G. HIV antiretroviral therapy in Africa. CCDR. 2018;44:2. 10.14745/ccdr.v44i02a06 PMC5864409 29770102

[3] Dillon DG, Gurdasani D, Riha J, Ekoru K, Asiki G, Mayanja BN, et al. Association of HIV and ART with cardiometabolic traits in sub-Saharan Africa: a systematic review and meta-analysis. International journal of epidemiology. 2013;42(6):1754–71. doi: 10.1093/ije/dyt198 24415610 PMC3887568

[4] Thienemann F, Sliwa K, Rockstroh JK. HIV and the heart: the impact of antiretroviral therapy: a global perspective. European heart journal. 2013;34(46):3538–46. doi: 10.1093/eurheartj/eht388 24126882

[5] Madeddu G, Fois A, Calia G, Babudieri S, Soddu V, Becciu F, et al. Chronic obstructive pulmonary disease: an emerging comorbidity in HIV-infected patients in the HAART era? Infection. 2013;41(2):347–53. doi: 10.1007/s15010-012-0330-x 22971938

[6] Deeks SG. HIV infection, inflammation, immunosenescence, and aging. Annual review of medicine. 2011;62:141–55. doi: 10.1146/annurev-med-042909-093756 21090961 PMC3759035

[7] Deeks SG, Lewin SR, Havlir DV. The end of AIDS: HIV infection as a chronic disease. The Lancet. 2013;382(9903):1525–33. doi: 10.1016/S0140-6736(13)61809-7 24152939 PMC4058441

[8] Sherman JJ, Davis L, Daniels K. Addressing the polypharmacy conundrum. US Pharm. 2017;42(6):14–20.

[9] Chireshe R, Naidoo K, Nyamakura R. Hypertension among human immunodeficiency virus infected patients on treatment at Parirenyatwa Hospital: A descriptive study. African journal of primary health care & family medicine. 2019;11(1):1–8. doi: 10.4102/phcfm.v11i1.1974 31478742 PMC6739546

[10] Organization WH. WHO global strategy on people-centred and integrated health services: interim report. World Health Organization; 2015.

[11] Guaraldi G, Malagoli A, Calcagno A, Mussi C, Celesia B, Carli F, et al. The increasing burden and complexity of multi-morbidity and polypharmacy in geriatric HIV patients: a cross sectional study of people aged 65–74 years and more than 75 years. BMC geriatrics. 2018;18(1):1–10. 29678160 10.1186/s12877-018-0789-0 PMC5910563

[12] Oni T, McGrath N, BeLue R, Roderick P, Colagiuri S, May CR, et al. Chronic diseases and multi-morbidity-a conceptual modification to the WHO ICCC model for countries in health transition. BMC public health. 2014;14(1):1–7. 24912531 10.1186/1471-2458-14-575 PMC4071801

[13] Adeyemi O, Lyons M, Njim T, Okebe J, Birungi J, Nana K, et al. Integration of non-communicable disease and HIV/AIDS management: a review of healthcare policies and plans in East Africa. BMJ global health. 2021;6(5):e004669. doi: 10.1136/bmjgh-2020-004669 33947706 PMC8098934

[14] Organization WH. WHO global strategy on people-centred and integrated health services: interim report. 2015. Google Scholar. 2019:1–50.

[15] Arksey H, O’Malley L. Scoping studies: towards a methodological framework. International Journal of Social Research Methodology. 2005;8(1):19–32.

[16] Castleberry A. NVivo 10 [software program]. Version 10. QSR International; 2012. AJPE; 2014.

[17] Osetinsky B, Genberg BL, Bloomfield GS, Hogan J, Pastakia S, Sang E, et al. Hypertension Control and Retention in Care Among HIV-Infected Patients: The Effects of Co-located HIV and Chronic Noncommunicable Disease Care. J Acquir Immune Defic Syndr. 2019;82(4):399–406. doi: 10.1097/QAI.0000000000002154 31658183 PMC6822379

[18] Ameh S. Evaluation of an integrated HIV and hypertension management model in rural south africa: a mixed methods approach. Glob Health Action. 2020;13(1):1750216. doi: 10.1080/16549716.2020.1750216 32316885 PMC7191904

[19] Ameh S, Klipstein-Grobusch K, D’Ambruoso L, Kahn K, Tollman SM, Gómez-Olivé FX. Quality of integrated chronic disease care in rural South Africa: user and provider perspectives. Health Policy Plan. 2017;32(2):257–66. doi: 10.1093/heapol/czw118 28207046 PMC5400067

[20] Birungi J, Kivuyo S, Garrib A, Mugenyi L, Mutungi G, Namakoola I, et al. Integrating health services for HIV infection, diabetes and hypertension in sub-Saharan Africa: a cohort study. 2021;11(11). 10.1136/bmjopen-2021-053412 PMC8565555 34728457

[21] Frieden M, Zamba B, Mukumbi N, Mafaune PT, Makumbe B, Irungu E, et al. Setting up a nurse-led model of care for management of hypertension and diabetes mellitus in a high HIV prevalence context in rural Zimbabwe: a descriptive study. BMC Health Serv Res. 2020;20(1):486. doi: 10.1186/s12913-020-05351-x 32487095 PMC7268639

[22] Gausi B, Berkowitz N, Jacob N, Oni T. Treatment outcomes among adults with HIV/non-communicable disease multimorbidity attending integrated care clubs in Cape Town, South Africa. AIDS Res Ther. 2021;18(1):72. doi: 10.1186/s12981-021-00387-3 34649586 PMC8515722

[23] Matima R, Murphy K, Levitt NS, BeLue R, Oni T. A qualitative study on the experiences and perspectives of public sector patients in Cape Town in managing the workload of demands of HIV and type 2 diabetes multimorbidity. PLoS One. 2018;13(3):e0194191. doi: 10.1371/journal.pone.0194191 29538415 PMC5851623

[24] Muddu M, Tusubira AK, Sharma SK, Akiteng AR, Ssinabulya I, Schwartz JI. Integrated hypertension and HIV care cascades in an HIV treatment program in Eastern Uganda: a retrospective cohort study. JJoaids 2019;81(5):552. 10.1097/QAI.0000000000002067 PMC6625912 31045649

[25] Patel P, Speight C, Maida A, Loustalot F, Giles D, Phiri S, et al. Integrating HIV and hypertension management in low-resource settings: Lessons from Malawi. 2018;15(3):e1002523. 10.1371/journal.pmed.1002523 PMC5841643 29513674

[26] Rabkin M, Palma A, McNairy ML, Gachuhi AB, Simelane S, Nuwagaba-Biribonwoha H, et al. Integrating cardiovascular disease risk factor screening into HIV services in Swaziland: lessons from an implementation science study. 2018;32(Suppl 1):S43. 10.1097/QAD.0000000000001889 PMC6309427 29952789

[27] Sando D, Kintu A, Okello S, Kawungezi PC, Guwatudde D, Mutungi G, et al. Cost-effectiveness analysis of integrating screening and treatment of selected non-communicable diseases into HIV/AIDS treatment in Uganda. J Int AIDS Soc. 2020;23 Suppl 1(Suppl 1):e25507. doi: 10.1002/jia2.25507 32562364 PMC7305460

[28] Shayo EH, Kivuyo S, Seeley J, Bukenya D, Karoli P, Mfinanga SG, et al. The Acceptability of Integrated Healthcare Services for HIV and Non-Communicable Diseases: Experiences from Patients and Healthcare Workers in Tanzania. 2021. 10.1186/s12913-022-08065-4 PMC9112557 35578274

[29] Wroe EB, Kalanga N, Dunbar EL, Nazimera L, Price NF, Shah A, et al. Expanding access to non-communicable disease care in rural Malawi: outcomes from a retrospective cohort in an integrated NCD-HIV model. BMJ Open. 2020;10(10):e036836. doi: 10.1136/bmjopen-2020-036836 33087368 PMC7580053

[30] Ameh S, Klipstein-Grobusch K, Musenge E, Kahn K, Tollman S, Gómez-Olivé FX. Effectiveness of an integrated approach to HIV and hypertension care in rural South Africa: controlled interrupted time-series analysis. Journal of acquired immune deficiency syndromes (1999). 2017;75(4):472. doi: 10.1097/QAI.0000000000001437 28640065 PMC5483981

[31] Rawat A, Uebel K, Moore D, Yassi A. Integrated HIV-Care into primary health care clinics and the influence on diabetes and hypertension care: an interrupted time series analysis in free state, South Africa over 4 years. JJJoAIDS 2018;77(5):476–83. doi: 10.1097/QAI.0000000000001633 29373391

[32] Topp SM, Chipukuma JM, Chiko MM, Matongo E, Bolton-Moore C, Reid SE. Integrating HIV treatment with primary care outpatient services: opportunities and challenges from a scaled-up model in Zambia. Health policy and planning. 2013;28(4):347–57. doi: 10.1093/heapol/czs065 22791556 PMC3697202

[33] Chamie G, Kwarisiima D, Clark TD, Kabami J, Jain V, Geng E, et al. Leveraging rapid community-based HIV testing campaigns for non-communicable diseases in rural Uganda. 2012. 10.1371/journal.pone.0043400 PMC3423366 22916256

[34] Govindasamy D, Kranzer K, van Schaik N, Noubary F, Wood R, Walensky RP, et al. Linkage to HIV, TB and non-communicable disease care from a mobile testing unit in Cape Town, South Africa. 2013;8(11):e80017. 10.1371/journal.pone.0080017 PMC3827432 24236170

[35] Kwarisiima D, Atukunda M, Owaraganise A, Chamie G, Clark T, Kabami J, et al. Hypertension control in integrated HIV and chronic disease clinics in Uganda in the SEARCH study. 2019;19(1):1–10. 10.1186/s12889-019-6838-6 PMC6501396 31060545

[36] Venables E, Edwards JK, Baert S, Etienne W, Khabala K, Bygrave H. "They just come, pick and go." The Acceptability of Integrated Medication Adherence Clubs for HIV and Non Communicable Disease (NCD) Patients in Kibera, Kenya. PLoS One. 2016;11(10):e0164634. doi: 10.1371/journal.pone.0164634 27764128 PMC5072644

[37] Kasaie P, Weir B, Schnure M, Dun C, Pennington J, Teng Y, et al. Integrated screening and treatment services for HIV, hypertension and diabetes in Kenya: assessing the epidemiological impact and cost-effectiveness from a national and regional perspective. J Int AIDS Soc. 2020;23 Suppl 1(Suppl 1):e25499. doi: 10.1002/jia2.25499 32562353 PMC7305418

[38] McCombe G, Murtagh S, Lazarus JV, Van Hout MC, Bachmann M, Jaffar S, et al. Integrating diabetes, hypertension and HIV care in sub-Saharan Africa: a Delphi consensus study on international best practice. BMC Health Services Research. 2021;21(1):1–9. 34781929 10.1186/s12913-021-07073-0 PMC8591882

