# Supplementary material for: Integrated chronic care models for people with comorbid of HIV and non-communicable diseases in Sub-Saharan Africa: A scoping review
Source: PLoS One. 2024 Mar 15;19(3):e0299904. doi: 10.1371/journal.pone.0299904 (PMC10942093; doi:10.1371/journal.pone.0299904)
Supplement: S1 File — (DOCX) [file pone.0299904.s001.docx]

| **Date of search** | **Database** | **Keywords** | **Number of publications retrieved** |
| --- | --- | --- | --- |
| 24/11/2021 | PubMed | ("HIV"[MeSH Terms] OR "HIV"[All Fields] OR ("acquired immunodeficiency syndrome"[MeSH Terms] OR ("acquired"[All Fields] AND "immunodeficiency"[All Fields] AND "syndrome"[All Fields]) OR "acquired immunodeficiency syndrome"[All Fields] OR "aids"[All Fields]) OR (("acquirable"[All Fields] OR "acquire"[All Fields] OR "acquired"[All Fields] OR "acquirement"[All Fields] OR "acquirements"[All Fields] OR "acquires"[All Fields] OR "acquiring"[All Fields]) AND ("human s"[All Fields] OR "humans"[MeSH Terms] OR "humans"[All Fields] OR "human"[All Fields]) AND ("immunologic deficiency syndromes"[MeSH Terms] OR ("immunologic"[All Fields] AND "deficiency"[All Fields] AND "syndromes"[All Fields]) OR "immunologic deficiency syndromes"[All Fields] OR ("immunodeficiency"[All Fields] AND "syndrome"[All Fields]) OR "immunodeficiency syndrome"[All Fields])) OR ("hiv"[MeSH Terms] OR "hiv"[All Fields] OR ("human"[All Fields] AND "immunodeficiency"[All Fields] AND "virus"[All Fields]) OR "human immunodeficiency virus"[All Fields])) AND ("noncommunicable diseases"[MeSH Terms] OR ("noncommunicable"[All Fields] AND "diseases"[All Fields]) OR "noncommunicable diseases"[All Fields] OR ("non"[All Fields] AND "communicable"[All Fields] AND "diseases"[All Fields]) OR "non communicable diseases"[All Fields] OR "ncds"[All Fields] OR "ncd"[All Fields] OR ("noncommunicable diseases"[MeSH Terms] OR ("noncommunicable"[All Fields] AND "diseases"[All Fields]) OR "noncommunicable diseases"[All Fields])) AND (("int j integr care"[Journal] OR ("integrated"[All Fields] AND "care"[All Fields]) OR "integrated care"[All Fields]) AND ("model"[All Fields] OR "models"[All Fields] OR "modelled"[All Fields] OR "modeler"[All Fields] OR "modeler s"[All Fields] OR "modelers"[All Fields] OR "modelling"[All Fields] OR "modelling"[All Fields] OR "modelization"[All Fields] OR "modelizations"[All Fields] OR "modelize"[All Fields] OR "modelized"[All Fields] OR "modelled"[All Fields] OR "modeller"[All Fields] OR "modellers"[All Fields] OR "modelling"[All Fields] OR "modellings"[All Fields] OR "models"[All Fields])) | 77 |
| 25/11/2021 | SCOPUS | TITLE-ABS-KEY (hiv OR human AND immunodeficiency AND virus AND non-communicable OR noncommunicable AND disease AND integration AND model ) | 27 |
| 25/11/2021 | EBSCOHost (Academic search complete, CINAHL, Health Resources, and PsychINFO) | SU (hiv or aids or acquired human immunodeficiency syndrome or human immunodeficiency virus) AND ( non-communicable diseases or ncds or ncd or noncommunicable diseases ) AND integrated care model | 13 |
| 26/11/2021 | Web of Science | (hiv or aids or acquired human immunodeficiency syndrome or human immunodeficiency virus) AND (non-communicable diseases or ncds or ncd or noncommunicable diseases) AND integrated care model | 101 |
| 28/11/2021 | Cochrane Library: | 10 Trials matching (hiv or aids or acquired human immunodeficiency syndrome or human immunodeficiency virus) AND (non-communicable diseases or ncds or ncd or noncommunicable diseases) AND integrated care model in Title Abstract Keyword | 10 |
| 30/11/2021 | Google Scholar | (hiv or aids or acquired human immunodeficiency syndrome or human immunodeficiency virus) AND (non-communicable diseases or ncds or ncd or noncommunicable diseases) AND integrated care model | 871 |
|  | **Total** | | 1099 |
|  | **Number of duplicates**  977 | | |
|  | **The number for abstract screening**  122 | | |

**Supplementary file 1:** Database searches
